# The anti-dysenteric drug fraxetin enhances anti-tumor efficacy of gemcitabine and suppresses pancreatic cancer development by antagonizing STAT3 activation

**DOI:** 10.18632/aging.203301

**Published:** 2021-07-28

**Authors:** Yangyang Guo, Yanyi Xiao, Hangcheng Guo, Hengyue Zhu, Dong Chen, Jilong Wang, Junjie Deng, Junjie Lan, Xiaodong Liu, Qiyu Zhang, Yongheng Bai

**Affiliations:** 1 Key Laboratory of Diagnosis and Treatment of Severe Hepato-Pancreatic Diseases of Zhejiang Province, The First Affiliated Hospital of Wenzhou Medical University, Wenzhou 325000, China; 2 The Sixth People’s Hospital of Wenzhou City, Wenzhou 325000, China; 3 Engineering Research Center of Clinical Functional Materials and Diagnosis and Treatment Devices of Zhejiang Province, Wenzhou Institute, University of Chinese Academy Sciences, Wenzhou 325000, China; 4 State Key Laboratory of Cellular Stress Biology, Innovation Center for Cell Signaling Network, School of Life Sciences, Xiamen University, Xiamen 361000, China; 5 Platform for Radiation Protection and Emergency Preparedness, School of Public Health and Management, Wenzhou Medical University, Wenzhou 325000, China; 6 Center for Health Assessment, Wenzhou Medical University, Wenzhou 325000, China; 7 Department for Hepatopancreatobiliary Surgery, The First Affiliated Hospital of Wenzhou Medical University, Wenzhou 325000, China

**Keywords:** fraxetin, pancreatic ductal adenocarcinoma, gemcitabine, STAT3, oxidative stress

## Abstract

Fraxetin, a natural product isolated and purified from the bark of *Fraxinus bungeana A.DC.*, has anti-inflammatory, analgesic, and anti-dysenteric activities. This study aimed to investigate the anti-tumor effects of fraxetin in pancreatic ductal adenocarcinoma (PDA). The effects of fraxetin on the malignant biological behavior of PDA were evaluated. Besides, the effects of fraxetin on the sensitivity of PCCs to gemcitabine, angiogenesis, the epithelial-mesenchymal transition (EMT), glucose metabolism, reactive oxygen species (ROS), and STAT3 activity were analyzed. By reversing the EMT, fraxetin suppressed proliferation, invasion, and migration, and induced mitochondrial-dependent apoptosis in PCCs. Also, treatment with fraxetin inhibited PDA growth and metastasis in nude mouse models. Furthermore, fraxetin made PCCs more sensitive to the chemotherapy drug gemcitabine. Mechanically, fraxetin treatment suppressed oncogenic KRAS-triggered STAT3 activation in PCCs and PDA tissues. Fraxetin shows significant interactions with STAT3 Src Homology 2 (SH2) domain residues, thereby preventing its homo-dimer formation, which then blocks the activation of downstream signal pathways. The anti-tumor activity of fraxetin in PDA was functionally rescued by a STAT3 activator colivelin. As a result, fraxetin hindered hypoxia-induced angiogenesis by decreasing HIF-1α and VEGFA expression, controlled glucose metabolism by reducing GLUT1 expression, inhibited the EMT by blocking the Slug-E-cadherin axis, and drove ROS-mediated apoptosis by regulating the STAT3-Ref1 axis. In conclusion, fraxetin enhances the anti-tumor activity of gemcitabine and suppresses pancreatic cancer development by antagonizing STAT3 activation.

## INTRODUCTION

Pancreatic ductal adenocarcinoma (PDA) is a highly malignant digestive system tumor with an abysmal prognosis and high mortality [1]. However, the underlying mechanism of PDA development remains unclear [2]. Surgery is the most common treatment for PDA. Only a few PDA patients, however, can receive surgery. Patients who cannot undergo surgery currently receive a combination of radiation and chemotherapy drugs, such as gemcitabine [3]. These adjuvant treatments can slow down the progression of PDA to some extent, but whether the patients can extend their lives requires further clinical observations. [4]. As a result, new treatments and effective drugs for PDA are urgently needed.

The Janus kinase-2 (JAK2)/signal transducer and activator of transcription-3 (STAT3) signaling pathway are involved in cellular proliferation, differentiation, apoptosis, and migration, among other physiological processes [5]. In response to a variety of cytokines or related factors (e.g., interferon, interleukins), JAK2 protein is activated via phosphorylation at two adjacent tyrosine residues and then phosphorylates and activates cytoplasmic STAT3 protein. Activated STAT3 can transfer into the nucleus and bind specific regulatory sequences to either activate or suppress transcription of target genes like c-Myc and cyclin D1 [6–9]. As a result, activated STAT3 plays a crucial role in regulating the cell cycle, apoptosis, and angiogenesis [10]. A series of studies have shown that in human PDA tissues the STAT3 is overactivated [11, 12], which may be caused by oncogenic KRAS mutation [13]. In addition, STAT3 controls several downstream oncogenic signaling pathways and is required for triggering pancreatic intraepithelial neoplasia progression to an invasive PDA [13]. Thus, targeting STAT3 could be an effective strategy for treating PDA progression.

Fraxetin is a plant-derived coumarin reported to have antibacterial, neuroprotective, anti-inflammatory, and anti-dysenteric properties. It is primarily isolated from *Fraxinus bungeana A.DC*. [14]. Recently, several studies have demonstrated that fraxetin suppresses tumor growth and metastasis in multiple cancer types [15, 16]. Notably, STAT3 was involved in fraxetin-mediated inhibitory effects on non-small-cell lung cancer cells proliferation [17]. Therefore, considering aberrant activation of STAT3 in PDA, we hypothesized that fraxetin might have the potential to protect against PDA progression by suppressing STAT3 activity.

This study aimed to investigate the effects of fraxetin on PDA *in vitro* and *in vivo* and further elucidated the underlying molecular mechanism. Besides, the effects of fraxetin on the sensitivity of PCCs to the chemotherapy drug gemcitabine were evaluated. Moreover, angiogenesis, glucose metabolism, reactive oxygen species (ROS), the epithelial-mesenchymal transition (EMT), and STAT3 activity were analyzed. Our results revealed that fraxetin enhanced the anti-tumor efficacy of gemcitabine and suppressed oncogenesis and the development of PDA by antagonizing STAT3 activation.

## MATERIALS AND METHODS

### Cell culture and drug treatment

**Figure 1:**
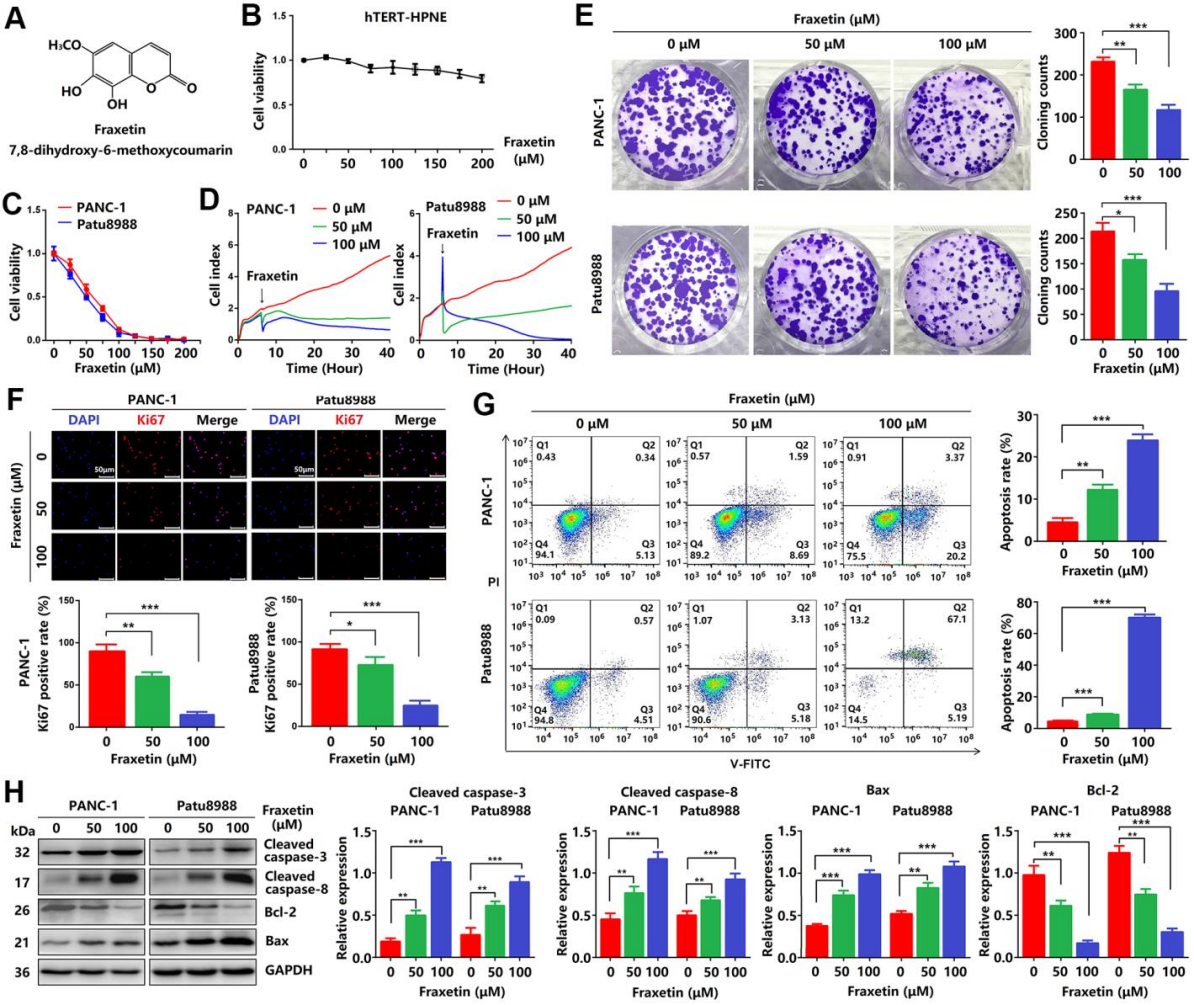
**Fraxetin inhibits cellular proliferation and induces mitochondrial-dependent apoptosis in PCCs.** (**A**) The chemical structure of fraxetin. (**B**, **C**) The CCK-8 assay was used to assess the viability of hTERT-HPNE, PANC-1 and Patu8988 cells with or without fraxetin treatment (0~200 μM). (**D**) Real-time cellular analysis (RTCA) was conducted to evaluate the growth of PANC-1 and Patu8988 cells with and without fraxetin treatment. (**E**) The colony formation assay was used to analyze the proliferation of PANC-1 and Patu8988 cells with and without fraxetin treatment. (**F**) Immunocytochemical staining of Ki67 in PANC-1 and Patu8988 cells with or without fraxetin treatment. Bar = 50 μm. (**G**) Flow cytometry analysis for cell apoptosis in PANC-1 and Patu8988 cells with or without fraxetin treatment. (**H**) Western blot analysis showing the expression of cleaved caspase-8, cleaved caspase-3, Bcl-2, and Bax in PANC-1 and Patu8988 cells with or without fraxetin treatment. Data were presented as the mean ± standard deviation, and were analyzed by One-way ANOVA with Bonferroni’s post-hoc test. ^**^P < 0.01, ^***^P < 0.001.

Human PCC lines PANC-1 and Patu8988, which have strong proliferative and invasive abilities [18], were provided from the Cell Bank of the Chinese Academy of Sciences (Shanghai, China). Dulbecco’s Modified Eagle Medium (DMEM, Invitrogen, USA) was used to culture cells, which was supplemented with 100 μg ml^-1^ streptomycin (Invitrogen), 100 U ml^-1^ penicillin, and 10% fetal bovine serum (FBS, Invitrogen). PANC-1 and Patu8988 cells were plated at a density of 1 × 10^6^ and preincubated for 24 h at 37° C, in the complete medium containing 5% FBS at approximately 70%-80% confluence in culture plates. Before drug treatment, the complete medium was replaced with a serum-free medium for 24 h. PANC-1 and Patu8988 cells were treated with 50 or 100 μM fraxetin (Figure 1A, CAS#: 574-84-5, Purity: ≥98% by HPLC, Yuanye Biotechnology, Shanghai, China) with or without colivelin (CAS#: 867021-83-8, MedChemExpress, NJ, USA).

### Cell counting kit-8 assay

Firstly, cells were plated in DMEM medium for 24 h followed by incubation in 96-well plates. Secondly, incubated cells were added to the plates at a density of 5 × 10^3^ cells/well and then treated with different fraxetin concentrations for 24 h. At appropriate time points, 10 μl of this reagent was added per well and incubated for another 2 h. Finally, the absorbance at 450 nm was calculated. All experiments were repeated at least in triplicate.

### Cell apoptosis analysis

PANC-1 and Patu8988 cells were cultured for 24 h in DMEM with different concentrations of fraxetin before being collected by centrifugation. 5 μl Annexin V-FITC (Multi Science, Hangzhou, China) and 5 μl propidium iodide (PI, Multi Science) were added to resuspended cells at room temperature and incubated for 15 minutes in the dark for apoptosis analysis. Finally, flow cytometry (BD Biosciences, USA) was used to examine cell apoptosis.

### Real-time cellular analysis

In a cell culture E16-Plate (ACEA Biosciences, San Diego, CA, USA), cells were seeded at a density of 4 × 10^3^ cells/well, and the cellular growth index was automatically recorded by the Label-free Real-time Cellular Analysis System (RTCA; Germany).

### Transwell invasion assay

The transwell inserts were coated with 150 μl Matrigel at 37° C for 2 h. Cells (4 × 10^5^) were collected and resuspended in a serum-free medium supplemented with fraxetin. Then cells were incubated in the upper chamber, and the lower chamber was infused with 500 μl DMEM containing 10% FBS. The plate was incubated at 37° C for 24 h, and then removed the gel and cells in the upper chamber. After formalin fixation, crystal violet (Sigma-Aldrich, USA) was used to stain the membrane for 10 min. Finally, using a microscope to obtain the image.

### Colony formation assay

Cells were plated in 6-well plates at a density of 1 × 10^3^ cells/well for 24 h, and then were treated with fraxetin. After treatment for 24 h, the culture medium was placed with DMEM for about 14 days. For 30 min, colonies were fixed in formaldehyde and then stained with crystal violet (Sigma-Aldrich). Finally, using a microscope (Leica Microsystems), cell colonies were counted.

### Wound healing assay

Cells were seeded in 6-well plates and cultured at 37° C for 24 h. To create a linear gap between the cells, the culture area was scratched with a crystal pipette tip. After that, the detached cells were rinsed in PBS and different quantities of fraxetin were applied. After allowing the cells to fill the gap, images of the culture area were captured under a microscope.

### Immunocytochemical staining

The immunofluorescence staining was carried out as described in our prior research [19]. Firstly, PANC-1 and Patu8988 cells treated with fraxetin were grown on glass coverslips for 24 h, and then fixed with 4% formaldehyde for 30 min. Secondly, cells were permeabilized with 0.1% Triton X-100, and blocked in 4% normal goat serum for 1.5 h. Cells were incubated overnight at 4° C with primary antibodies followed by incubation at room temperature with secondary antibody (1:400) for 1 h. Finally, cells were stained with DAPI (Beyotime Biotechnology, China), and images were captured under a fluorescence microscope.

### Glucose metabolism assay

A Seahorse XF96 Extracellular Flux Analyser (Seahorse Bioscience, North Billerica, MA, USA) was used to quantify the intact cellular oxygen consumption rate (OCR) and extracellular acidification rate (ECAR). In short, 1.0 ×10^4^ of PANC-1 and Patu8988 cells were seeded into 96-well cell plates and incubated at 37° C with 5% CO2 overnight. For 24 hours, both cells were treated with or without different concentrations of fraxetin. Meanwhile, the calibration plates were incubated in a non-CO2 incubator overnight at 37° C. After the probe calibration was finished, both cell mediums were replaced with assay medium, and the cell plate replaced the probe plate. The analyzer plotted the value of OCR followed by injection of the compounds sequentially as follows: oligomycin (inhibitor of ATP synthase; 2.5 μM), Carbonyl cyanide 4-(trifluoromethoxy) phenylhydrazone (FCCP, an uncoupler of OXPHOS; 2 μM), rotenone (inhibitor of complex I; 0.25 μM) and anti-mildew A (inhibitor of complex III; 0.25 μM) (n = 8). Continuous injections of glucose (10 mM), oligomycin (1 μM), and 2-Deoxyglucose (2-DG, 50 mM) were used to assess ECAR (n = 8). Following the completion of the test, the BCA Protein Assay Kit was used to quantify protein concentration to normalize OCR and ECAR, as directed by the manufacturer.

### Nude mouse tumorigenicity

Male nude mice (BALB/c) weighing 20-22 g and 6-8 weeks old were obtained from the Experimental Animal Centre of Wenzhou Medical University (Wenzhou, China). All mice were housed in a humidity-, temperature-, and light-controlled environment. Mice were divided into two groups at random, with four mice in each group. Then the right thigh root of mice was injected subcutaneously with 3 × 10^6^ PANC-1 cells. The experimental group received intragastric administration of fraxetin (25 mg/kg·d) every 3 days for a month. In contrast, the control group got DMSO intragastrically. Tumor volume was determined using the formula V = (length width^2^)/2, with the length being the tumor’s longest dimension. All of the mice were euthanized by CO2 asphyxiation at the end of the experiment. This animal study was approved by the Institutional Animal Care and Use Committee of Wenzhou Medical University, China. The animal experiments were conducted according to all regulatory institutional guidelines for animal welfare (National Institutes of Health Publications, NIH Publications No. 80-23) [20].

### Histopathological analysis

Tumor specimens from animals embedded in paraffin were cut into 4-μm slices and stained with hematoxylin and eosin (HE, Yuanye Biotechnology, Shanghai, China). According to a previous report [18] Immunohistochemical (IHC) analysis was performed. Briefly, 4-μm thick sections were dewaxed with xylene and then rehydrated in ethanol sequentially. Antigen retrieval was performed with sections in 0.1 percent sodium citrate buffer (pH 6.0), and endogenous peroxidase activity was inhibited with 3% hydrogen peroxide (Beyotime). Anti-Ki67 (1:200, Abcam) and anti-E-cadherin (1:200, Abcam) were used as primary antibodies for IHC staining. Image-Pro Plus software (version 6.0, Media Cybernetics, Silver Spring, MD, USA) was used to calculate the integrated optical density (IOD). All samples were blindly analyzed semi-quantitatively or quantitatively by two independent investigators.

### Western blot analysis

Whole proteins from the normal pancreatic ductal cell (hTERT-HPEN) and PCCs (PANC-1, Patu8988, and BxPc-3) or PDA tissues treated with fraxetin for 24 h were collected, and protein concentrations were determined by double star choline acid protein analysis kit (Beyotime). SDS-PAGE was used to separate total protein (20 g) from each sample, then transferred to a polyvinylidene difluoride membrane. (PVDF, Solarbio, Beijing, China). After blocking the membranes for 1 h at room temperature with 5% skim nonfat milk, membranes were incubated with the primary antibodies (1:1000), including anti-caspase-3 (Proteintech, Wuhan, China), anti-caspase-8 (Proteintech), anti-Bcl-2 (Proteintech), anti-Bax (Proteintech), anti-N-cadherin (Abcam), anti-Slug (Abcam), anti-JAK2 (Abcam), anti-p-JAK2 (Abcam), anti-STAT3 (CST), anti-p-STAT3 (CST), anti-E-cadherin (Abcam), anti-type I collagen (Abcam), anti-GLUT1 (Affinity Biosciences, Cincinnati, OH, USA), anti-VEGFA (Affinity), anti-HIF-1α (Affinity), anti-Ref1 (Abcam), and anti-vimentin (Abcam), overnight at 4° C. After washing in TBST (10 mM Tris-HCl, 150 mM NaCl and 0.1% Tween-20), membranes were incubated with secondary antibodies (1:400) for 1 h at room temperature. Finally, the protein bands were visualized using chemiluminescence detection on autoradiographic film and the GADPH antibody (1:8000, Proteintech) was used as the internal reference.

### ROS level assay

ROS level assay was performed as directed by the manufacturer (Beyotime). In brief, PANC-1 or Patu8988 cells were seeded in a 6-well plate with a density of about 5×10^4^/well. After the cells adhere to the wall, cells were treated with fraxetin for 24 h. On the second day, 2 ml of the dilution (DCFH-DA, 1:1000, Beyotime) was added to each well, incubated at 37° C for 30 min, and then the cells were collected. Images were captured using a fluorescence microscope (Leica Microsystems). In addition, the cells were also resuspended in PBS, and ROS level was detected by flow cytometry (BD Biosciences).

### Database analysis

The GEPIA 2 database website (http://gepia2.cancer-pku.cn/#analysis) was used to examine STAT3 expression in PDA tissues and adjacent normal tissues. Besides, it was also used to evaluate the correlation between STAT3 expression and KRAS activity. Moreover, the potential target candidates were analyzed using the PharmMapper Server (http://www.lilab-ecust.cn/pharmmapper/index.html) according to a previous report [21].

### Molecular docking

Fraxetin structure was downloaded from PubChem (https://pubchem.ncbi.nlm.nih.gov) as SDF files and converted into mol2 format using Chimera. The STAT3 crystal structure was downloaded from PDB no. 6NJS as a model (http://www.rcsb.org). Compound and protein were converted into PDBQT format using AudoDockTools and docked using AutoDock Vina. Visualize conformations and interactions were prepared by PyMol, Chimera, and LigPlot+.

### Statistical analysis

Data were presented as the means ± standard deviations. GraphPad Prism (version 8.0, GraphPad Software, Inc., La Jolla, CA, USA) was used to conduct all statistical analyses. The differences between the two groups were analyzed using a two-sided Student’s t-test. When there were more than two groups, a one-way analysis of variance (ANOVA) with Bonferroni’s post-hoc test was performed. It was determined that P<0.05 was statistically significant.

## RESULTS

### Fraxetin inhibits cellular proliferation and induces mitochondrial-dependent apoptosis in PCCs

Firstly, CCK-8 assay was performed to investigate the effects of fraxetin on PCC proliferation. As shown in Figure 1B, fraxetin (0~200 μM) was less toxic to hTERT-HPNE cells. While the viability of PANC-1 cells incubated for 24 h gradually decreased when the concentration of fraxetin gradually increased in a concentration-dependent manner. Similarly, the effects of fraxetin on Patu8988 cells were confirmed by CCK-8 results (Figure 1C), especially when the concentration of fraxetin is 25~100 μM. Thus, we selected 50 and 100 μM of fraxetin for subsequent anti-proliferative studies. Based on the RTCA results, fraxetin treatment significantly slowed the growth of PCCs, and the effect was time-dependent (Figure 1D). Moreover, fraxetin significantly reduced the number of colony formation in PANC-1 and Patu8988 cells (Figure 1E). These results supported the anti-proliferative activity of fraxetin in PCCs.

A previous study has shown that Ki67 (also known as MKI67) is a proliferation-related cellular marker that is highly expressed in PDA tissues [22]. In this study, fraxetin-treated PCCs had a lower ratio of Ki67-positive cells in total cells than control cells (Figure 1F). These findings suggested that fraxetin inhibits PCC proliferation and that this activity is time- and concentration-dependent.

Given fraxetin has anti-proliferative activity in PCCs, whether fraxetin can induce cellular apoptosis. The apoptotic PCCs were examined by flow cytometry to detect labeled Annexin V-FITC/PI. As expected, we found that fraxetin concentration-dependently increased the ratio of early apoptotic cells but also enhanced the ratio of late apoptotic and necrotic cells (Figure 1G). Caspases, a family of cysteine proteases, are the central regulators of apoptosis. As a key protease, caspase-3 is cleaved and activated during the early stage of apoptosis to execute apoptosis by cleaving targeted cellular proteins [23]. In this study, the expression of cleaved caspase-3 was upregulated by fraxetin treatment (Figure 1H). Besides, fraxetin also increased the expression of cleaved caspase-8 (Figure 1H), which can be used as an apoptotic initiator to activate caspase-3 [24]. Moreover, reduced Bcl-2 expression and enhanced Bax expression in fraxetin-treated PCCs were observed (Figure 1H). Collectively, these findings indicated that fraxetin induces apoptosis of PCCs through a mitochondrial-dependent pathway.

### Fraxetin inhibits PCC invasion and migration via Slug-E-cadherin-dependent EMT

**Figure 2:**
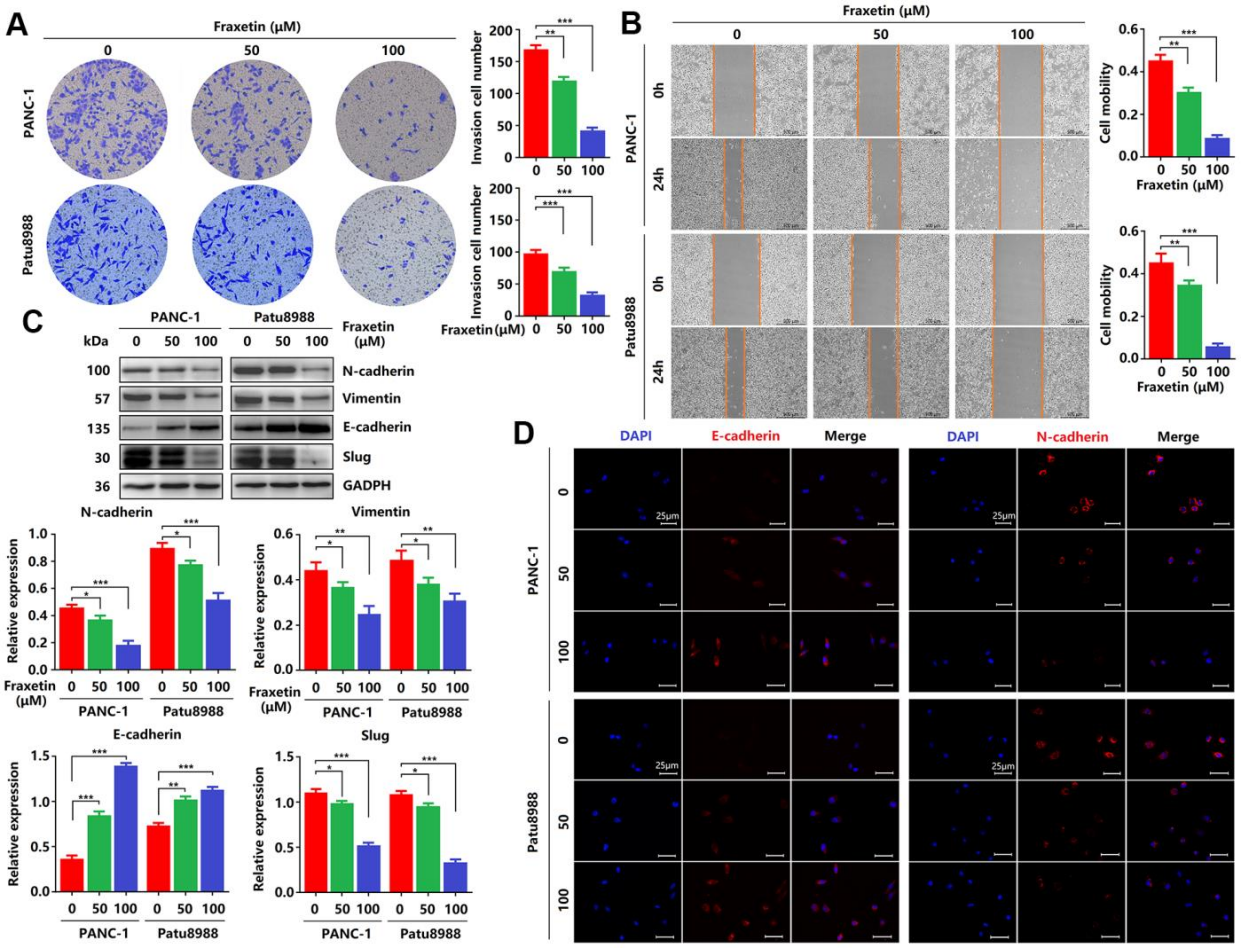
**Fraxetin inhibits PCC invasion and migration by regulating the Slug-E-cadherin axis.** (**A**) Transwell chamber assays were used to investigate the effects of fraxetin on the invasion number of PANC-1 and Patu8988 cells. (**B**) A wound healing assay was used to determine the effects of fraxetin on the migration rate of PANC-1 and Patu8988 cells. (**C**) N-cadherin, Vimentin, E-cadherin, and Slug expression in fraxetin-treated PANC-1 and Patu8988 cells as shown on Western blot. (**D**) Immunocytochemical staining of E-cadherin and α-SMA in fraxetin-treated PANC-1 and Patu8988 cells. Bar = 25 μm. Data were presented as the mean ± standard deviation and were analyzed by One-way ANOVA with Bonferroni’s post-hoc test. ^*^P < 0.05, ^**^P < 0.01, ^***^P < 0.001.

Next, we evaluated the effects of fraxetin on the invasion and migration of PCCs by transwell invasion and wound healing assay. As shown in Figure 2A, in PANC-1 and Patu8988 cells, fraxetin treatment significantly reduced the number of invasive cells in a concentration-dependent manner. In addition, the migration of PCCs was inhibited by fraxetin, and it is concentration-dependent (Figure 2B). Further research revealed that the suppressive effect of fraxetin on the invasion and migration of PCCs might be related to the inhibition of epithelial-mesenchymal transition (EMT). EMT is a developmental process that plays a vital role in PDA progression and metastasis [25]. The EMT is characterized by the loss of cell-to-cell adhesion and is associated with the phenotypic conversion of epithelial cells into mesenchymal-like cells [26]. E-cadherin is the primary adhesion protein associated with epithelial cells and is considered an active suppressor of invasion and growth of PDA [27]. Currently, EMT-associated transcription factor Slug (Snail2), a member of the Snail superfamily, is known to bind directly to the E-boxes of E-cadherin gene promoter and repress its transcription [28]. Thus, we evaluated the expression of E-cadherin and Slug in fraxetin-treated PCCs. E-cadherin expression was enhanced in PCCs upon fraxetin treatment (Figure 2C, 2D). In addition, fraxetin decreased the expression of matrix components, including N-cadherin and Vimentin (Figure 2C, 2D). Moreover, fraxetin reduced Slug expression (Figure 2C). Thus, these data reveal an inhibitory effect of fraxetin on the invasion and migration of PCCs by regulating the EMT process dependent on the Slug-E-cadherin axis.

### Fraxetin inhibits tumor growth and metastasis of PDA in animal xenograft models

**Figure 3:**
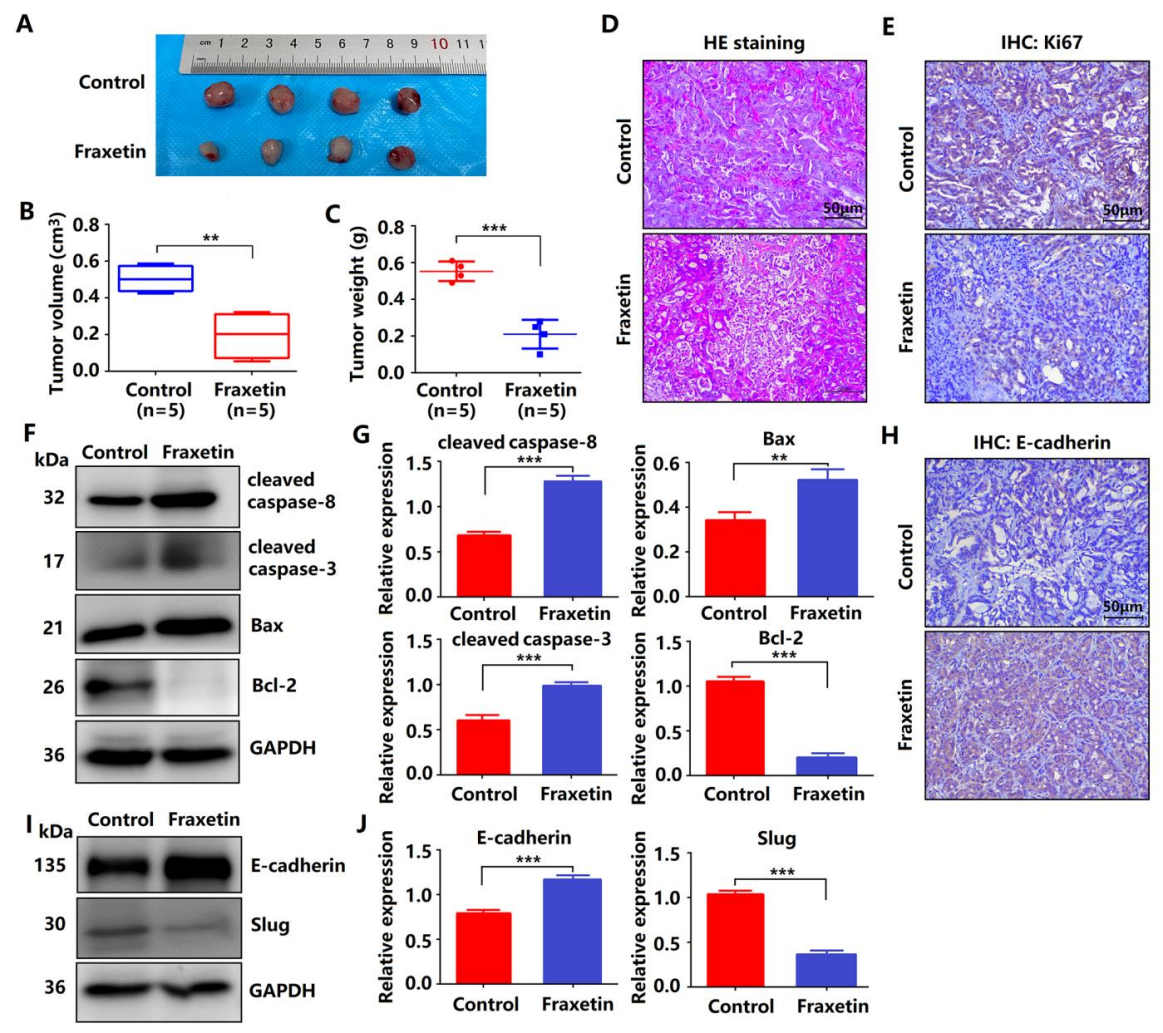
**Fraxetin inhibits tumor growth and metastasis of PDA in animal xenograft models.** (**A**) Effects of fraxetin on experimental groups’ morphologic changes. (**B**) Effect of fraxetin on the volume of tumors in animal xenograft models. (**C**) Effects of fraxetin on tumor weight. (**D**) PDA pathological results in tissues of the model group stained with HE. Bar = 50 μm. (**E**) Immunohistochemical (IHC) staining for Ki67 in fraxetin-treated models. Bar = 50 μm. (**F**, **G**) Western blot analysis of cleaved caspase-8, cleaved caspase-3, Bax, and Bcl-2 expression in PANC-1 and Patu8988 cells treated with or without fraxetin. (**H**) IHC staining for E-cadherin in fraxetin-treated models. Bar = 50 μm. (**I**, **J**) Western blot analysis of E-cadherin and Slug expression in PANC-1 and Patu8988 cells treated with or without fraxetin. Data were presented as the mean ± standard deviation, and were analyzed by a two-sided Student’s t-test. ^*^P < 0.05, ^**^P < 0.01, ^***^P < 0.001.

Given fraxetin inhibited the proliferation, migration and invasion, and induced apoptosis of PANC-1 and Patu8988 cells *in vitro*, whether the similar anti-tumor mechanism of fraxetin occurred in nude mice of PDA xenografts *in vivo*. Fraxetin (25 mg/kg·d) was administered continuously for 30 days to the mice subjected to the injection of PCCs. The experimental groups underwent morphologic changes as a result of fraxetin treatment, as shown in Figure 3A. The administration of fraxetin significantly reduced the tumor’s volume and weight (Figure 3B, 3C). Evidence from HE-staining showed PDA pathological results in tissues of the model group (Figure 3D). In addition, in fraxetin-treated models, the decreased proportion of Ki67-positive cells in total cells indicated tumor cell proliferation inhibition (Figure 3E). Further research revealed that fraxetin treatment increased the expression of cleaved caspase-8 and cleaved caspase-3 (Figure 3F, 3G). Moreover, fraxetin upregulated Bax expression and downregulated Bcl-2 expression (Figure 3F, 3G). As a result, our *in vivo* experiments revealed that fraxetin induced PCC apoptosis via mitochondria. To access whether fraxetin suppressed PDA metastasis, we examined the expression of EMT-related proteins. Immunohistochemical analysis showed that fraxetin significantly increased E-cadherin expression (Figure 3H). The enhanced expression of E-cadherin was confirmed by Western Blot results (Figure 3I, 3J). Furthermore, fraxetin reduced the expression of Slug expression (Figure 3I, 3J). Thus, fraxetin inhibited PDA metastasis in animal xenograft models via Slug-E-cadherin-dependent EMT.

### Fraxetin enhances the sensitivity of PCCs to gemcitabine

**Figure 4:**
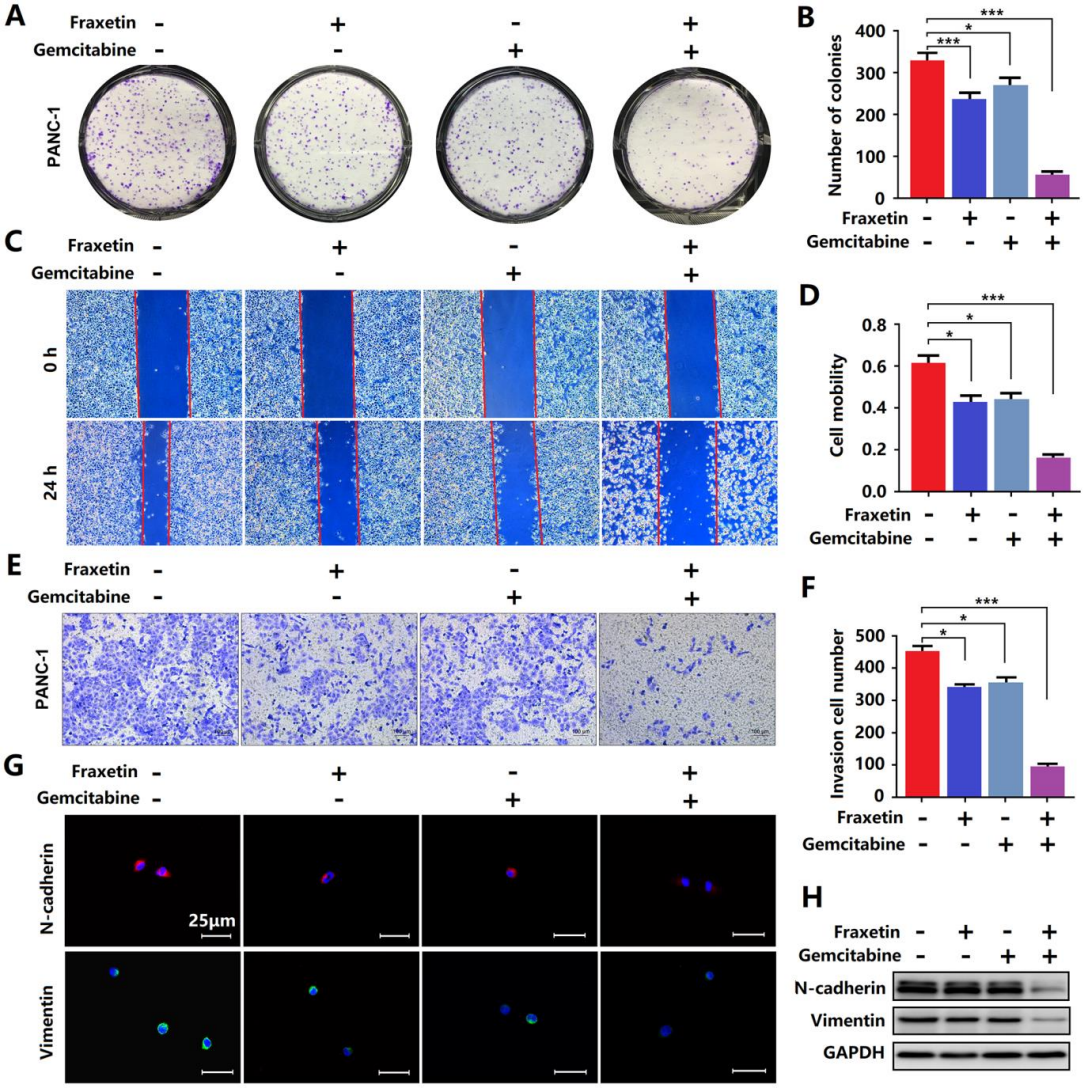
**Fraxetin enhanced the sensitivity of PCCs to gemcitabine.** (**A**, **B**) By using a colony formation assay, the effects of fraxetin on the proliferation of gemcitabine-treated PANC-1 cells were investigated. (**C**, **D**) A wound healing assay was used to determine the effects of fraxetin on the migrated rate of gemcitabine-treated PANC-1 cells. (**E**, **F**) The transwell chamber assay was used to examine the effects of fraxetin on the invasion number of gemcitabine-treated PANC-1 cells. (**G**) Immunocytochemical staining of N-cadherin and Vimentin in gemcitabine-treated PANC-1 with or without fraxetin treatment. Bar = 50 μm. (**H**) N-cadherin and Vimentin expression in gemcitabine-treated PANC-1 with or without fraxetin treatment, as determined by Western blot analysis. Data were presented as the mean ± standard deviation, and were analyzed by One-way ANOVA with Bonferroni’s post-hoc test and two-sided Student’s t-test. ^*^P < 0.05, ^***^P < 0.001.

Gemcitabine is currently the first-line drug for PDA chemotherapy, but chemotherapy resistance is widespread and has become the main reason for the failure of PDA chemotherapy [29]. Therefore, it is crucial to inhibit gemcitabine resistance and promote its anti-PDA efficacy. Here, evidence from colony formation assay showed that gemcitabine has a particular inhibitory effect on the proliferation of PCCs, and this effect can be enhanced by fraxetin (Figure 4A, 4B). Besides, fraxetin treatment also strengthens the inhibitory effects of gemcitabine on the infiltration and invasion of PCCs (Figure 4C–4F) by downregulating N-cadherin and Vimentin expression (Figure 4G, 4H). Thus, these findings indicated that fraxetin could enhance the sensitivity of PCCs to the chemotherapy drug gemcitabine.

### Fraxetin induces STAT3 inactivation by occupying its SH2 domain *in vitro* and *in vivo*

**Figure 5:**
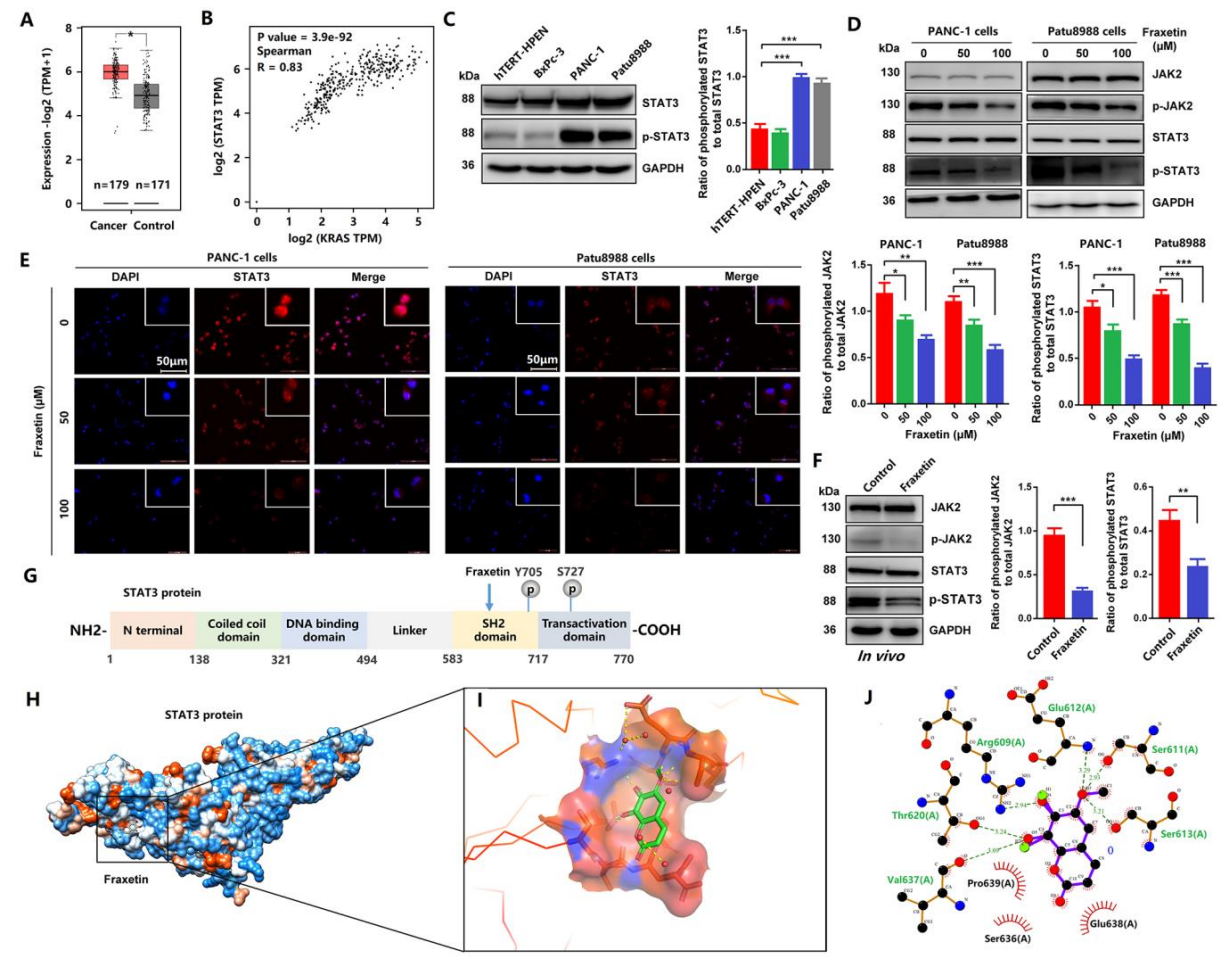
**Fraxetin induced STAT3 inactivation by occupying its SH2 domain *in vitro* and *in vivo*.** (**A**) The expression of STAT3 in PDA tissues and adjacent normal tissues in the GEPIA 2 database was analyzed. (**B**) The correlation between STAT3 expression and KRAS activity in the GEPIA 2 database was evaluated. (**C**) The expression and phosphorylation of STAT3 in a normal pancreatic ductal cell (hTERT-HPEN^KRAS(-)^) and PCCs (PANC-1^KRAS G12D^, Patu8988^KRAS G12V^ and BxPc-3^KRAS(-)^) (**D**) JAK2 and STAT3 expression and phosphorylation in PANC-1 and Patu8988 cells with and without fraxetin treatment, as seen on a Western blot. (**E**) Immunocytochemical staining of STAT3 in PANC-1 and Patu8988 cells with or without fraxetin treatment. Bar = 50 μm. (**F**) Western blot analysis showing the expression and phosphorylation of JAK2 and STAT3 in fraxetin-treated animal xenograft models. The overview of Fraxetin binding in the STAT3 SH2 domain by using UCSF chimera. (**G**) Sequence analysis indicates that the STAT3 protein harbor conserved motifs, such as the DNA binding region and SH2 domain. (**H**) The overview of Fraxetin binding in the STAT3 SH2 domain by using UCSF chimera. (**I**) 3D representation of fraxetin binding sites in the STAT3 SH2 domain (yellow dotted line means hydrogen bond) by using PyMol. (**J**) Detailed 2D representation of fraxetin binding sites in STAT3 SH2 domain residues interactions generated by LigPlot plus. Data were presented as the mean ± standard deviation, and were analyzed by One-way ANOVA with Bonferroni’s post-hoc test and two-sided Student’s t-test. ^*^P < 0.05, ^**^P < 0.01, ^***^P < 0.001.

A previous study has shown that STAT3 is involved in fraxetin-mediated inhibition in the proliferation of lung cancer cells [17]. In PDA, KRAS mutation, including G12D and G12V, drives STAT3 activation [13], consistent with our conclusion (Figure 5A–5C). Moreover, the over-activation of STAT3 induces the chemoresistance of PCCs to the chemotherapy drug gemcitabine [30]. Thus, we hypothesized that fraxetin exerts its protective effects by targeting STAT3 activity in PANC-1 (KRAS G12D) and Patu8988 (KRAS G12V) cells. Although there was no significant difference in the expression of JAK2 and STAT3 protein between the fraxetin-treated group and the control group, the phosphorylation levels of JAK2 (Y1007) and STAT3 (Y705) were significantly suppressed by fraxetin treatment (Figure 5D). Considering that the activation of STAT3 is due to the nuclear expression of phosphorylated STAT3, we detected the nuclear localization of STAT3 by immunocytochemical staining. The results showed that the nuclear expression of STAT3 in PANC-1 and Patu8988 cells was inhibited by fraxetin (Figure 5E). Furthermore, in animal xenograft models, fraxetin administration suppressed the phosphorylation of JAK2 and STAT3 (Figure 5F). Taken together, our *in vitro* and *in vivo* experiments showed that inhibition of STAT3 activity might be critical for fraxetin-mediated anti-tumor effects in PDA.

Next, we analyzed the interaction between fraxetin and STAT3 protein. As shown in Figure 5G, the STAT3 protein harbor conserved motifs, including Src Homology 2 (SH2) domain. We found interactions between fraxetin and protein residues of STAT3 (6NJS) crystal structure (Figure 5H). Fraxetin shows significant interactions with STAT3 SH2 domain residues (Figure 5I). It makes hydrogen bonds (green dotted line) with Arg609, Ser611, glu612, Ser613, Thr620, and Val637 residues (Figure 5I, 5J). It also creates a good group of hydrophobic pocket (red symbol) formed by Ser636, Glu638, and Pro639 (Figure 5J). As a result, fraxetin has placed at the center SH2 domain to occupy the pTyr-recognition site of its SH2 domain of another STAT3 monomer, thereby preventing its homo-dimer formation, which then inhibits the phosphorylation of STAT3 and blocks the activation of STAT3-downstream signaling pathways.

### Reactivation of STAT3 reverses the anti-tumor effects of fraxetin in PDA

**Figure 6:**
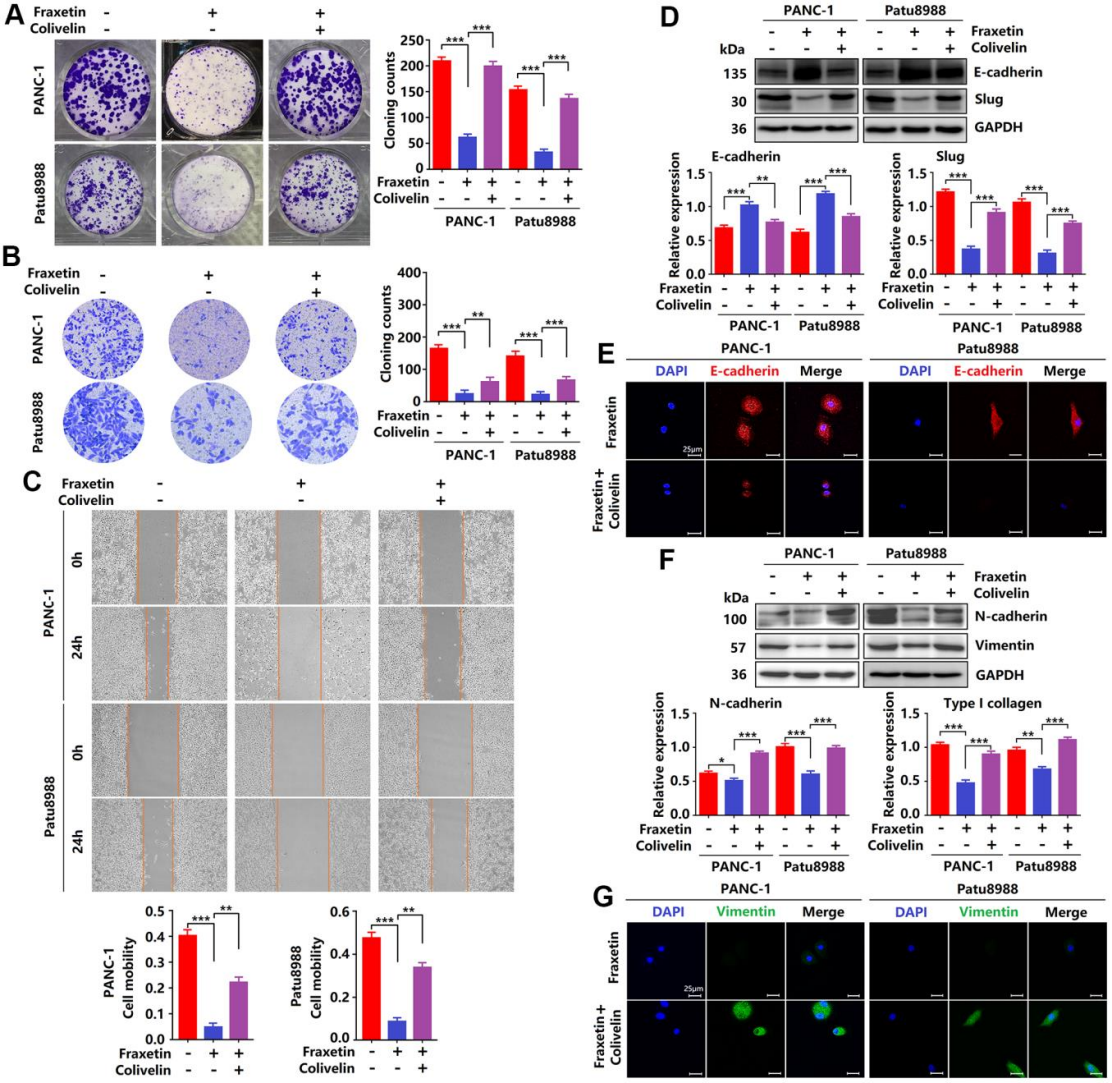
**Reactivation of STAT3 reverses the anti-tumor effects of fraxetin.** (**A**) The proliferation of fraxetin-treated PANC-1 and Patu8988 cells with or without colivelin treatment was analyzed by colony formation assay. (**B**) The transwell chamber assay analyzed the invasion ability of fraxetin-treated PANC-1 and Patu8988 cells with or without colivelin treatment. (**C**) A wound healing assay was used to determine the migration ability of fraxetin-treated PANC-1 and Patu8988 cells with or without colivelin treatment. (**D**) E-cadherin and Slug expression in fraxetin-treated PANC-1 and Patu8988 cells with and without colivelin treatment, as determined by Western blot analysis. (**E**) Immunocytochemical staining of E-cadherin in fraxetin-treated PANC-1 and Patu8988 cells with or without colivelin treatment. Bar = 25 μm. (**F**) N-cadherin and Type I collagen expression in fraxetin-treated PANC-1 and Patu8988 cells with and without colivelin treatment, as determined by Western blot analysis. (**G**) Immunocytochemical staining of vimentin in fraxetin-treated PANC-1 and Patu8988 cells with or without colivelin treatment. Bar = 25 μm. Data were presented as the mean ± standard deviation, and were analyzed by One-way ANOVA with Bonferroni’s post-hoc test and two-sided Student’s t-test. ^*^P < 0.05, ^**^P < 0.01, ^***^P < 0.001.

Since fraxetin exerts its protective effects on PDA by targeting STAT3 activity, whether reactivation of STAT3 can rescue the inhibitory effects of fraxetin. In this study, colivelin, a neuroprotective peptide with brain permeability, was used to activate STAT3 [31]. Firstly, evidence from colony formation assay showed that colivelin increased the number of colonies, revealing that reactivated STAT3 relieved fraxetin-mediated inhibition of PCC proliferation (Figure 6A). In addition, colivelin enhanced the number of invasive cells in fraxetin-treated PANC-1 and Patu8988 cells, indicating that colivelin-induced overactivation of STAT3 reversed fraxetin-mediated inhibition of PCC invasion (Figure 6B). Furthermore, the ability of cell mobility in fraxetin-treated PCCs was strengthened by colivelin treatment, suggesting that upregulated STAT3 activity by colivelin abolished the inhibition of PCC migration caused by fraxetin (Figure 6C). Thus, these results confirmed again that reactivation of STAT3 by colivelin enhances the biological behavior of PCCs upon fraxetin treatment, and the STAT3 is crucial for the inhibitory effect of fraxetin on PDA.

Next, we evaluated the effects of STAT3 reactivation on the EMT. Colivelin reduced E-cadherin expression in fraxetin-treated PCCs, as determined by Western blot and immunocytochemical staining. (Figure 6D, 6E), revealing that epithelial phenotypic features are suppressed by reactivation of STAT3. Further study showed that Slug expression in fraxetin-treated PANC-1 and Patu8988 cells was increased by colivelin treatment (Figure 6D), thereby indicating that STAT3 reactivation abolished fraxetin-mediated EMT inhibition. Moreover, in fraxetin-treated PCCs, colivelin upregulated the expression of N-cadherin and Vimentin (Figure 6F, 6G), suggesting that STAT3 reactivation induced excessive accumulation of matrix. Collectively, STAT3 reactivation by colivelin reverses the anti-tumor effects of fraxetin on PDA.

### Fraxetin inhibits angiogenesis, glucose metabolism and EMT in PCCs

**Figure 7:**
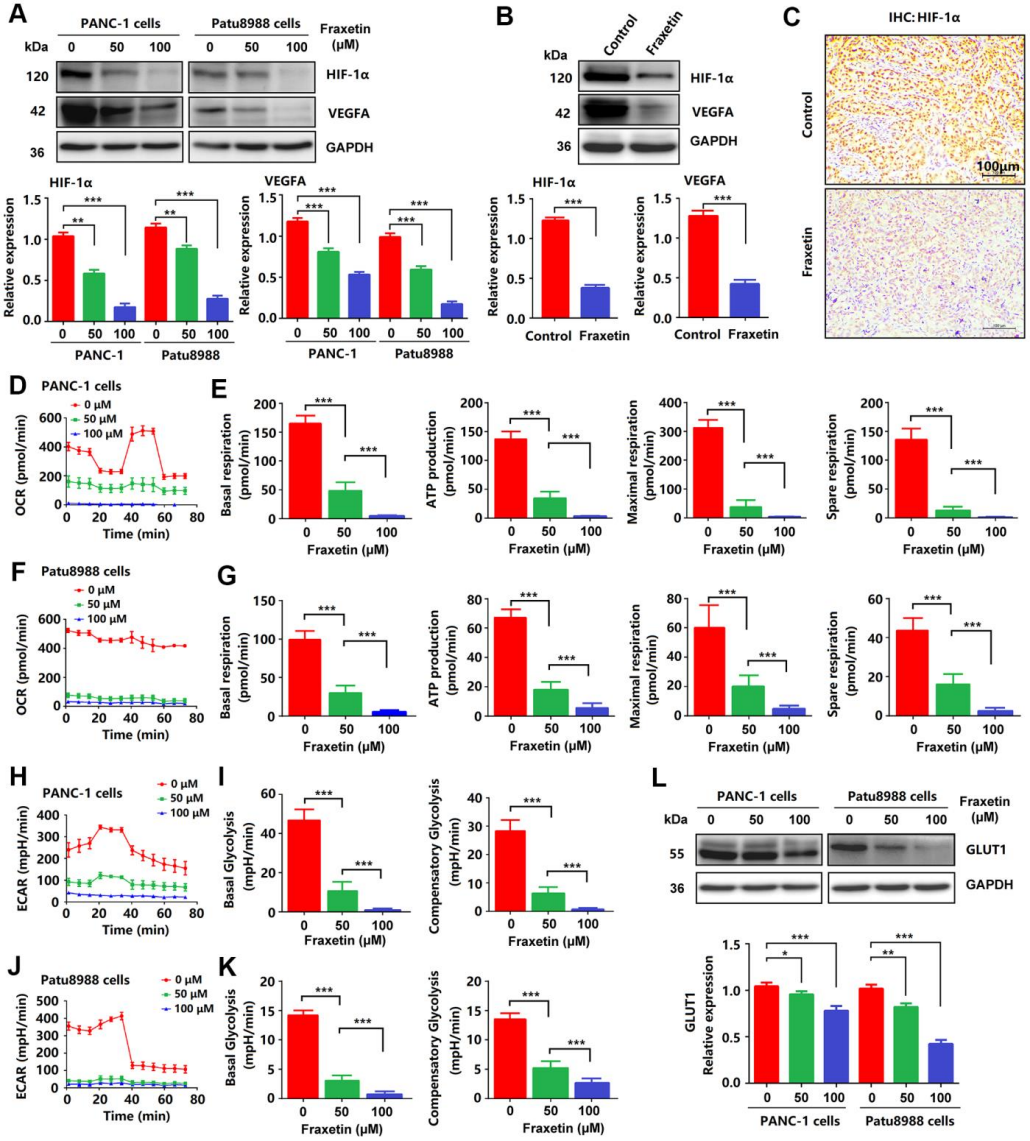
**Fraxetin inhibited angiogenesis, glucose metabolism and EMT in PCCs.** (**A**) HIF-1 and VEGFA expression in fraxetin-treated PANC-1 and Patu8988 cells as seen on a Western blot. (**B**) Western blot analysis of HIF-1 and VEGFA expression in fraxetin-treated animal xenograft models. (**C**) IHC staining for HIF-1α in fraxetin-treated models. Bar = 100 μm. (**D**–**G**) Glucose metabolism assay shows downregulated oxygen consumption rate (OCR), basal respiration, spare respiration, maximal respiration, and ATP production in fraxetin-treated PANC-1 and Patu8988 cells. (**H**–**K**) Glucose metabolism assay showing reduced levels of extracellular acidification rate (ECAR), basal glycolysis and compensatory glycolysis in fraxetin-treated PANC-1 and Patu8988 cells. (**L**) GLUT1 expression in fraxetin-treated PANC-1 and Patu8988 cells as seen on a Western blot. Data were presented as the mean ± standard deviation, and were analyzed by One-way ANOVA with Bonferroni’s post-hoc test and two-sided Student’s t-test. ^*^P < 0.05; ^**^P < 0.01, ^***^P < 0.001.

To further clarify the mechanism by which fraxetin exerts its protective effects on PDA by inhibiting STAT3 activation, we analyzed the expression and release of downstream target molecules of this signaling. STAT3 downstream molecules, including Bcl-2, E-cadherin, hypoxia-inducible factor-1α (HIF-1α), and vascular endothelial growth factor-α (VEGFA), are involved in the regulation of cellular proliferation, apoptosis, angiogenesis, and metastasis [10, 32]. As mentioned before, reduced Bcl-2 expression in fraxetin-treated PCCs indicated mitochondrial pathway-dependent apoptosis (Figure 1G). Also, fraxetin inhibits the EMT process by blocking the Slug-E-cadherin axis (Figure 2C, 2D). In addition to these, fraxetin-mediated downregulation in the expression levels of HIF-1α and VEGFA revealed the inhibition of hypoxia-induced angiogenesis (Figure 7A–7C). Moreover, fraxetin treatment reduced the oxygen consumption rate (OCR) in PANC-1 and Patu8988 cells by inhibiting basal respiration, spare respiration, maximal respiration, and ATP production. (Figure 7D–7G). The levels of extracellular acidification rate (ECAR) were decreased by fraxetin by antagonizing basal glycolysis and compensatory glycolysis (Figure 7H–7K). Importantly, reduced expression of glucose transporter type 1 (GLUT1), a uniporter protein that facilitates the transport of glucose across the plasma membranes of mammalian cells for metabolism [33], in fraxetin-treated PCCs was responsible for the reduction of glucose metabolism (Figure 7L). Thus, these findings indicated that in PDA fraxetin hinders hypoxia-induced angiogenesis by decreasing the expression levels of HIF-1α and VEGFA, controlling glucose uptake and metabolism by reducing GLUT1 expression, and inhibiting EMT by blocking the Slug-E-cadherin axis.

### Fraxetin drives ROS-mediated apoptosis by regulating the STAT3-Ref1 axis

**Figure 8:**
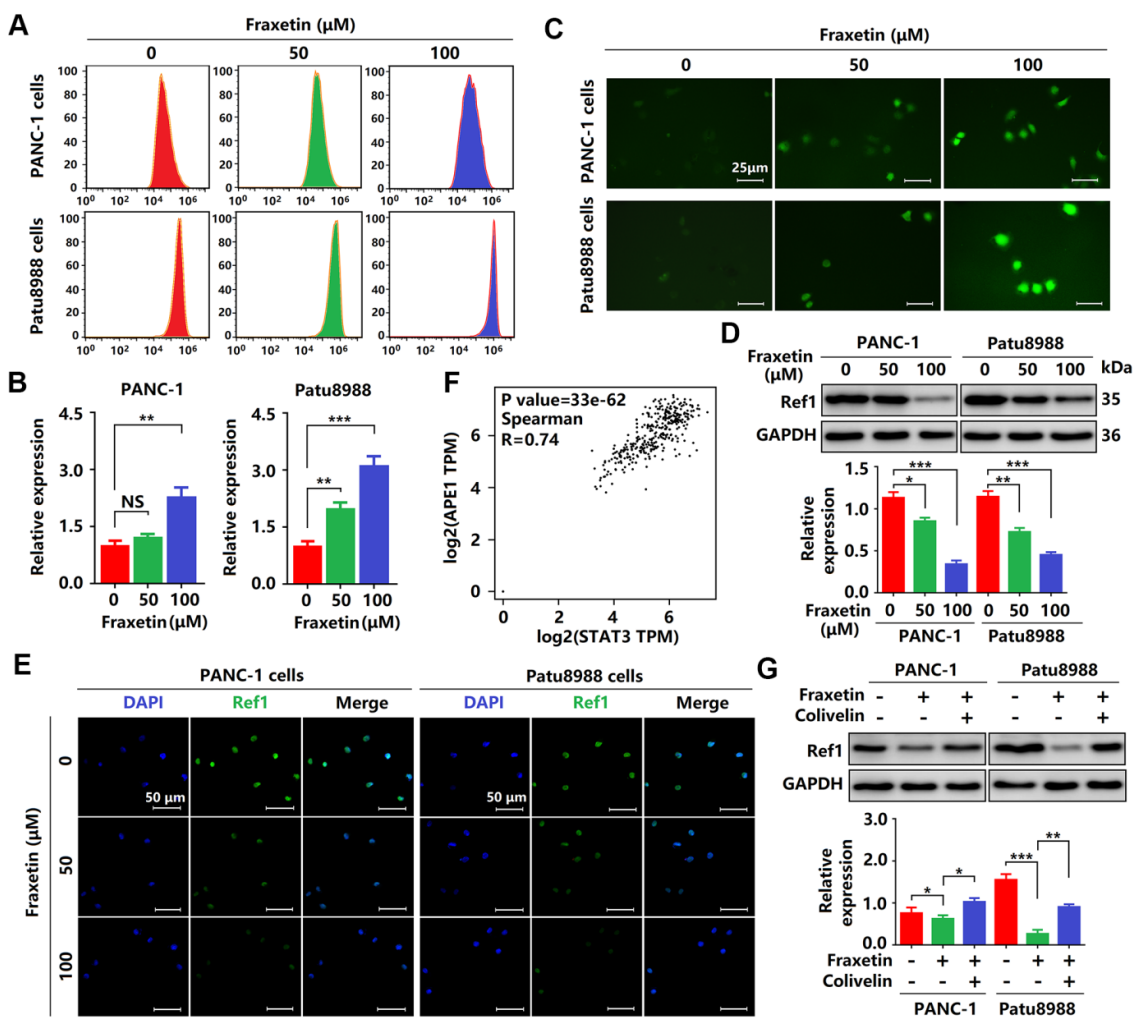
**Fraxetin drove ROS-mediated apoptosis by regulating the STAT3-Ref1 axis.** (**A**, **B**) Flow cytometric analysis showing the levels of ROS in fraxetin-treated PANC-1 and Patu8988 cells. (**C**) Immunocytochemical staining showing the levels of ROS in fraxetin-treated PANC-1 and Patu8988 cells. Bar = 25 μm. (**D**) Ref1 expression in fraxetin-treated PANC-1 and Patu8988 cells as seen on a Western blot. (**E**) Immunocytochemical staining showing Ref1 expression in fraxetin-treated PANC-1 and Patu8988 cells. Bar = 50 μm. (**F**) The correlation between STAT3 and APE1 (encoding Ref1 protein) in the GEPIA 2 database was evaluated. (**G**) Ref1 expression in fraxetin-treated PANC-1 and Patu8988 cells with or without colivelin treatment, as determined by Western blot analysis. Data were presented as the mean ± standard deviation, and were analyzed by One-way ANOVA with Bonferroni’s post-hoc test and two-sided Student’s t-test. ^*^P < 0.05; ^**^P < 0.01, ^***^P < 0.001.

As mentioned above, fraxetin induces cell apoptosis via the Bcl-2-mediated mitochondrial pathway. In PDA, Bcl-2 expression can be regulated by ROS [34]. In this study, we found that the levels of ROS were significantly increased in fraxetin-treated PCCs (Figure 8A–8C), suggesting that fraxetin-induced apoptosis might be mediated by ROS. Further study revealed that downregulated Ref1 expression was involved in ROS-triggered apoptosis of PCCs when exposed to fraxetin (Figure 8D, 8E). Ref1 is a vital target molecule of STAT3 that can regulate ROS levels [35]. We also found that Ref1 expression was positively associated with STAT3 activity in PDA (Figure 8F). Reactivation of STAT3 with colivelin abolished fraxetin-induced downregulation of Ref1 expression in PDA (Figure 8G). Thus, these results indicated that fraxetin drove ROS-mediated apoptosis by regulating the STAT3-Ref1 axis.

## DISCUSSION

In the present study, we investigated for the first time the anti-tumor effects of fraxetin on pancreatic ductal adenocarcinoma (PDA) *in vitro* and *in vivo*. In pancreatic cancer cells (PCCs), fraxetin inhibited cell proliferation by downregulating Ki67 expression. In addition, fraxetin induced apoptosis in PCCs via a mitochondrial-mediated pathway. Moreover, fraxetin suppressed the invasion and migration of PCCs by regulating epithelial-mesenchymal transition (EMT) dependent on the Slug-E-cadherin axis. In nude mouse models, PDA growth and metastasis were reduced by fraxetin treatment. Thus, these evidence supported that fraxetin exerts effective protection against PDA.

PDA, which accounts for more than 90% of pancreatic cancer cases, is a aggressive type of cancer with a 5-year survival rate of less than 8% [1]. PDA can be hard to treat surgically due to the organ’s location, and because the disease has often spread in the body by the time it is diagnosed. Thus, developing effective drugs for treating PDA is still needed. Recently, multiple studies show that several natural products may become novel candidate agents for developing PDA therapeutics [36, 37]. Fraxetin is a plant-derived coumarin primarily isolated from *Fraxinus bungeana A.DC.*, and has been reported to exert potent antibacterial, neuroprotective and anti-inflammatory effects [14]. In breast cancer cells, fraxetin inhibits cellular proliferation, induces mitochondrial-dependent apoptosis by upregulating Bax expression and downregulating Bcl-2 expression [15]. Also, fraxetin treatment causes a significant cell cycle arrest and pro-apoptotic effects in non-small-cell lung cancer cells [17]. Similar to the above studies, our *in vivo* and *in vitro* experiments also identified anti-tumor effects of fraxetin in PDA by inducing PCC apoptosis, inhibiting proliferation, and reducing tumor growth. Moreover, fraxetin prevented PDA progression by suppressing the invasion and migration of PCCs. Thus, our results provide a rationale for using fraxetin as a potential supplemental treatment for PDA.

Interestingly, we found that fraxetin can enhance the sensitivity of PCCs to the chemotherapy drug gemcitabine. Gemcitabine is accepted as the standard treatment for patients with locally or metastatic advanced PDA, but the problem of its drug resistance has become increasingly prominent [38]. How to solve the weakening of its anti-tumor effect due to drug resistance is an important task now. For this reason, drugs such as human recombinant hyaluronidase (PEGPH20) or CD40 monoclonal antibody (CP-870,893) have entered clinical studies to increase the efficacy of gemcitabine against advanced PDA [39, 40]. Our results identified fraxetin as an adjuvant drug to strengthen the anti-tumor effect of gemcitabine by inhibiting the EMT and PCC proliferation. Therefore, fraxetin may be a potential drug development candidate for PDA treatment.

Further studies have shown that STAT3 activity is responsible for fraxetin-mediated anti-tumor effects [11–13]. Although STAT3 is dispensable for the development of the pancreas, the majority of PDA show constitutive activation of STAT3 by phosphorylation at Y705 and induction into the nucleus [13]. Notably, STAT3 activation is mainly driven by oncogenic KRAS mutation, found in ~95% of pancreatic intraepithelial neoplasias (PanINs), the earliest pre-neoplastic stages of PDA progression [13, 41, 42]. Thus, targeted inhibition of STAT3 might be a potential therapeutic strategy for PDA. AG-409, a tyrosine kinase inhibitor that inhibits STAT3 activation, decreased the invasion and metastasis ability of PDA [43]. In addition, FLLL31 and FLLL32, 2 novel small-molecule STAT3 inhibitors, derived from curcumin, inhibited multiple oncogenic processes, induced apoptosis in PCC lines, and reduced tumor growth and vascularity PDA mouse xenografts [44]. Our findings also supported STAT3 as a critical target for the anti-tumor activity of fraxetin. Fraxetin treatment not only inhibited the phosphorylation (Y1007) of JAK2 in PCCs, but also antagonized the phosphorylation (Y705) and nuclear localization of STAT3. Furthermore, reactivating STAT3 with colivelin relieved fraxetin-mediated induction of apoptosis, and inhibition of proliferation, invasion and migration of PCCs, thereby enhancing the biological behavior of PCCs upon fraxetin treatment.

Significantly, fraxetin antagonized the phosphorylation (Y705) of STAT3 protein by direct interaction. Fraxetin binds to STAT3 SH2 domain residues by hydrogen bonding with Arg609, Ser611, glu612, Ser613, Thr620, and Val637 residues. It also creates a favorable group of hydrophobic pocket formed by Ser636, Glu638, and Pro639. Thus, fraxetin occupies the pTyr-recognition site of the STAT3 SH2 domain to prevent its homo-dimer formation, and thereby suppresses the phosphorylation of STAT3 and blocks the activation of STAT3-downstream signal pathways.

In PDA initiation and development, STAT3 exerts its important physiological function through a series of downstream target molecules. The STAT3-downstream molecules, including Bcl-2, E-cadherin, HIF-1α, and VEGFA, play critical roles in regulating proliferation, apoptosis, angiogenesis, and metastasis of PCCs [10, 32]. E-cadherin is a calcium-dependent cell adhesion protein involved in mechanisms regulating cell-cell adhesion and mobility of epithelial cells [45, 46]. Loss of intercellular adhesion and increased cell motility promotes tumor cell invasion and migration through EMT [47]. In the hypoxic microenvironment of PDA, HIF-1α is crucial for angiogenesis by inducing VEGFA expression [48]. In addition, enhanced HIF-1α activity in PDA promotes glycolysis by regulating GLUT1 expression in PCCs [49]. Our findings indicated that fraxetin reduced Bcl-2 expression and thereby induced mitochondrial-dependent apoptosis in PCCs. Also, fraxetin inhibited the EMT process by blocking the Slug-E-cadherin axis. Moreover, fraxetin hindered hypoxia-induced angiogenesis by decreasing the expression levels of HIF-1α and VEGFA, controlled glucose uptake and metabolism by reducing GLUT1 expression. Significantly, reactivation of STAT3 with colivelin reversed fraxetin-mediated inhibition of EMT via the Slug-E-cadherin axis. These evidence supported a crucial role of STAT3 in fraxetin-mediated anti-tumor effects again.

In addition to the direct regulation of Bcl-2 expression by STAT3, we also found that fraxetin-induced apoptosis was mediated indirectly by ROS. ROS triggers the mitochondrial apoptosis pathway by regulating Bcl-2 expression [34]. Further study revealed that reduced Ref1 expression contributed to ROS-triggered apoptosis of PCCs when exposed to fraxetin. As a target molecule of STAT3, Ref1 plays an essential role in the regulation of ROS levels and oxidative stress [35]. Colivelin was used to reactivate STAT3 and thereby resulted in the abolishment of fraxetin-induced downregulation of Ref1 expression in PDA. These results indicated that fraxetin drove ROS-mediated apoptosis by regulating the STAT3-Ref1 axis.

However, there exist some apparent limitations. First, a genetic approach to evaluate the role of STAT3 in fraxetin-mediated anti-tumor effects needs to be presented. In addition, the mechanism of KRAS-mutation (G12D or G12V) affects the anti-tumor effects of fraxetin should be clarified. It is also worth mentioning that the anti-tumor potential of fraxetin in clinical application, including pharmacokinetics and its side effects, needs to be further clarified.

**Figure 9:**
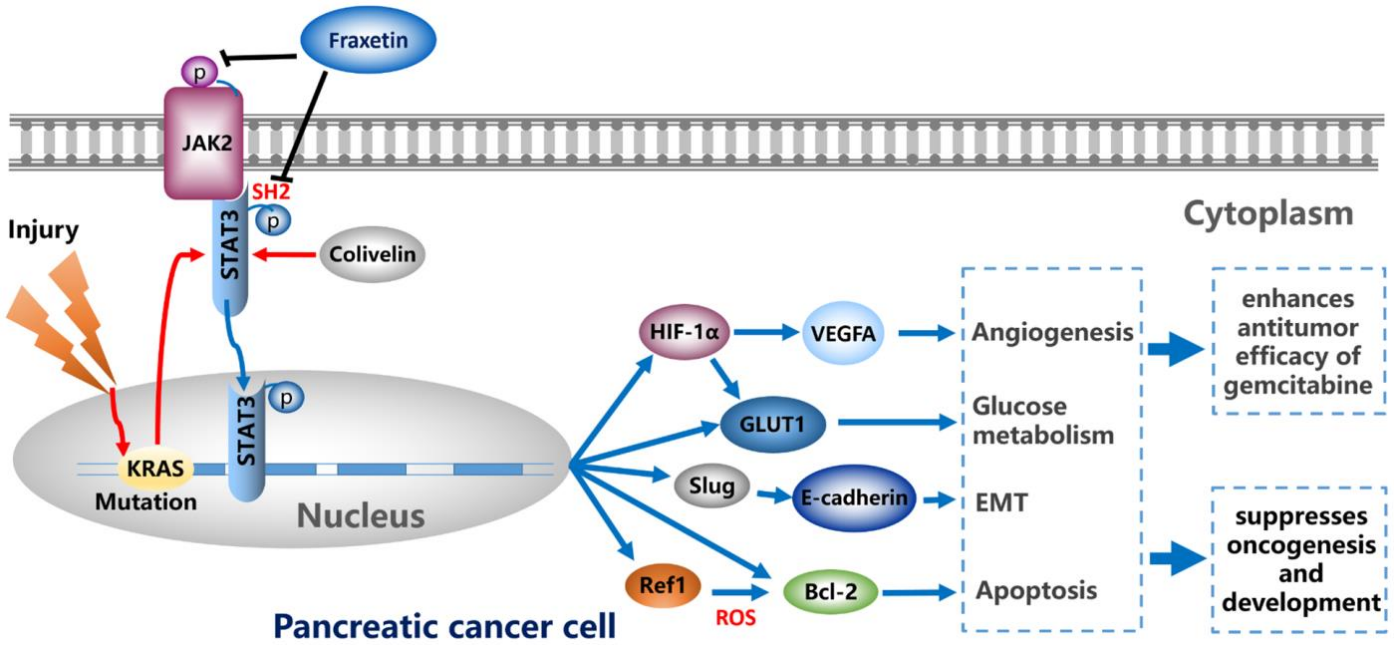
Fraxetin enhances the anti-tumor efficacy of gemcitabine and suppresses the oncogenesis and development of PDA via antagonizing STAT3 activation.

In conclusion, by regulating EMT, fraxetin suppressed PCC proliferation, induced mitochondrial-mediated apoptosis, and inhibited invasion and migration. Fraxetin treatment inhibited PDA growth and metastasis in nude mouse models. Moreover, fraxetin strengthens the anti-tumor effect of gemcitabine by inhibiting PCC proliferation and EMT. Mechanically, oncogenic KRAS-triggered STAT3 activation in PCCs and PDA tissues was suppressed by fraxetin via occupying STAT3 SH2 domain. Consequently, fraxetin hindered hypoxia-induced angiogenesis by decreasing HIF-1α and VEGFA expression, controlled glucose metabolism by reducing GLUT1 expression, inhibited the EMT by blocking the Slug-E-cadherin axis, and drove ROS-mediated apoptosis by regulating STAT3-Ref1 axis. Reactivation of STAT3 reverses the anti-tumor effects of fraxetin (Figure 9). Thus, pharmacological inhibition of STAT3 activity with fraxetin suppresses the oncogenesis and development of PDA by antagonizing STAT3 activation, and fraxetin can be a therapeutic approach to PDA.
